# Ensuring General Data Protection Regulation Compliance and Security in a Clinical Data Warehouse From a University Hospital: Implementation Study

**DOI:** 10.2196/63754

**Published:** 2025-04-17

**Authors:** Christine Riou, Mohamed El Azzouzi, Anne Hespel, Emeric Guillou, Gouenou Coatrieux, Marc Cuggia

**Affiliations:** 1 University Hospital of Rennes, 2 rue Henri Le Guilloux, Rennes, 35000, France, 33 0299284215; 2 DOMASIA, LTSI, UMR INSERM, University of Rennes, Rennes, France; 3 IMT Atlantique Bretagne Pays de Loire, Brest, France

**Keywords:** clinical data warehouse, privacy, personal data protection, legislation, security, compliance, personal data, applicability, experiential analysis, university hospitals, French, France, data hub, operational challenge

## Abstract

**Background:**

The European Union’s General Data Protection Regulation (GDPR) has profoundly influenced health data management, with significant implications for clinical data warehouses (CDWs). In 2021, France pioneered a national framework for GDPR-compliant CDW implementation, established by its data protection authority (Commission Nationale de l’Informatique et des Libertés). This framework provides detailed guidelines for health care institutions, offering a unique opportunity to assess practical GDPR implementation in health data management.

**Objective:**

This study evaluates the real-world applicability of France’s CDW framework through its implementation at a major university hospital. It identifies practical challenges for its implementation by health institutions and proposes adaptations relevant to regulatory authorities in order to facilitate research in secondary use data domains.

**Methods:**

A systematic assessment was conducted in May 2023 at the University Hospital of Rennes, which manages data for over 2 million patients through the eHOP CDW system. The evaluation examined 116 criteria across 13 categories using a dual-assessment approach validated by information security and data protection officers. Compliance was rated as met, unmet, or not applicable, with criteria classified as software-related (n=25) or institution-related (n=91).

**Results:**

Software-related criteria showed 60% (n=15) compliance, with 28% (n=7) noncompliant or partially compliant and 12% (n=3) not applicable. Institution-related criteria achieved 72% (n=28) compliance for security requirements. Key challenges included managing genetic data, implementing automated archiving, and controlling data exports. The findings revealed effective privacy protection measures but also highlighted areas requiring regulatory adjustments to better support research.

**Conclusions:**

This first empirical assessment of a national CDW compliance framework offers valuable insights for health care institutions implementing GDPR requirements. While the framework establishes robust privacy protections, certain provisions may overly constrain research activities. The study identifies opportunities for framework evolution, balancing data protection with research imperatives.

## Introduction

Health data, once primarily recorded on paper, are now predominantly digital worldwide. The rise of artificial intelligence (AI) methods has amplified the value of these digital data across academic and industrial sectors globally. Countries around the world, including France, the United Kingdom, Germany, and the United States, are implementing strategies to structure, secure, and ethically process these data. For instance, the United Kingdom’s NHS Digital and Germany’s medical data initiatives are designed to centralize and facilitate data sharing, thereby stimulating research and innovation, particularly in AI algorithm development [1-3].

In the European Union, the European Health Data Space (EHDS) initiative aims to create a unified framework for health data access and exchange across member states. The EHDS will empower individuals by giving them greater control over their electronic personal health data and will support the reuse of these data for research, innovation, and policy-making, fostering a genuine single market for electronic health record systems and AI-based health technologies [4,5].

Similarly, France’s Health Data Hub centralizes health data to promote research and innovation, emphasizing the critical role played by clinical data warehouses (CDWs), particularly those within hospitals. These CDWs compile data generated during care for reuse, which is essential for achieving the ambitious goals set by these data strategies [6,7]. This approach is encouraged by public authorities, as evidenced by specific calls for projects in 2022 and 2023 [8]. The national ambition is to have large datasets to support extensive research projects. The University Hospital of Rennes, with its eHOP CDW [9], is a pioneer and has extended its use to other institutions [10].

However, these data, as personally identifiable information (PII), are subject to strict regulations, including the General Data Protection Regulation (GDPR) [11] and the French Data Protection Act (Loi Informatique et Libertés) [12]. Additionally, in 2021, the French data protection authority (Commission Nationale de l’Informatique et des Libertés; CNIL) published a specific framework for the processing of PII in the creation of CDWs [13], referred to as the CDW framework in this paper. While this regulatory framework ensures data security, it is also cumbersome to implement. Compliance with these stringent regulations is both a prerequisite and a necessity, but it poses significant challenges in practical application.

The aim of the GDPR is to protect citizens’ privacy in the context of widespread digitization while facilitating the circulation of data. It emphasizes transparency in processing health data and enhances patients’ control over their use, which are widely regarded as positive steps. However, the recent literature underscores several challenges in applying GDPR to the reuse of health data for research. It is often perceived as a barrier to data sharing due to burdensome administrative procedures [14-16] and strict requirements for preventing reidentification of individuals [15]. Moreover, discrepancies in interpretation among European Union member states lead to inconsistencies between national regulations, particularly in balancing privacy and solidarity [14,17]. Such disparities complicate the coordination of international research projects [14-18]. A survey conducted across 28 European countries found a slight preference among patients for direct control over their data rather than delegation to a scientific authority [18].

Proposals for implementing CDWs with privacy and security measures by design have been advanced in Ireland [19]. These include deidentification processes, stringent access controls, safeguards against cyberattacks, regulatory compliance, and governance structures defining authorized users and use parameters. However, these proposals remain theoretical and lack national-level recommendations. In France, the implementation of CDWs must align with the CNIL CDW framework, which sets rigorous standards to ensure GDPR compliance while enabling the seamless reuse of health data in a competitive environment.

To date, no other country has established comprehensive guidelines for CDW implementation, nor has any published an evaluation of the practical application of such measures. This paper addresses this gap by sharing insights from the deployment of the eHOP CDW in university hospitals (Centre Hospitalier Universitaire) across Western France.

The objective of this study is to assess the applicability of this pioneering framework. The evaluation criterion is the level of compliance of the eHOP CDW with the CNIL CDW framework. For requirements where compliance proves challenging, we propose adjustments for consideration by national authorities. This paper first outlines the defining features of CDWs and the regulatory requirements stemming from GDPR and the French Data Protection Act. It then introduces the CNIL CDW framework and the methodology for compliance assessment. Finally, we discuss the applicability of certain requirements in light of research challenges and propose alternative measures to facilitate the implementation of CDWs in health care institutions.

## Methods

### Ethical Considerations

The research reported in the paper was not a study on human participants, and no treatment of personal data was carried out during the research. The European GDPR does not apply to this research. The eHOP data warehouse was authorized by the French data protection authority (CNIL). Deliberation 2020-028 of February 27, 2020, authorized the University Hospital of Rennes to implement personal data processing for the purpose of a health data warehouse called “eHOP Rennes,” ensuring respect of patients' information and rights.

### Clinical Data Warehouses

A CDW is a repository of large health data already collected elsewhere, consolidated into a single database for reuse in various treatments whose purposes are not known in advance and differ from the initial purpose of collection. These data are stored for a long period.

The hospital CDW centralizes all health data from patients who visited the institution by extracting information from the hospital information system. The CDW is based on a robust technical architecture and an adapted data model, ensuring the reliability and relevance of the stored information. Before any use, the data undergo integration steps including pseudonymization, standardization, and validation.

To exploit the CDW, it is equipped with secure tools for targeting study populations, multicriteria queries, and data visualization. For each study, specific datasets called datamarts are generated.

Each CDW operates under a robust governance structure and strict access policies to ensure the security and ethical use of health data. In this study, we examine the eHOP CDW as a representative example. The development and implementation of eHOP CDW are the results of a collaborative effort between the University Hospital of Rennes, Institut National de la Santé et de la Recherche Médicale UMR 1099, and Enovacom, a subsidiary of the company Orange Healthcare, which specializes in digital health care solutions.

The deployment and maintenance of the eHOP CDW are managed by a dedicated team within each hospital. This multidisciplinary team comprises medical IT specialists, IT engineers, data scientists, and a data protection officer (DPO), ensuring compliance with data protection regulations and operational effectiveness. As of December 2024, the eHOP CDW at the University Hospital of Rennes holds health information on 2 million patients, represented across 167 million documents and including 1.3 billion data elements.

### Current Regulations

The GDPR governs the processing of personal data belonging to European citizens. Adopted in 2016, it replaced European Directive 95/46/EC [20] to address the challenges posed by emerging digital technologies. While directly applicable in all European Union member states, the regulation allows some flexibility for the introduction of supplementary national provisions. Citizens’ rights have been strengthened, particularly in terms of information and transparency regarding the processing of their personal data. New rights have also been introduced, such as the right to be forgotten (also referred to as the right to erasure).

The GDPR introduces key concepts such as “accountability” and “privacy by design,” aimed at increasing the accountability of stakeholders and ensuring that privacy is considered from the earliest stages of data processing operations. It also requires data controllers to conduct a Data Protection Impact Assessment for high-risk processing operations. The regulation is built on 6 fundamental principles: lawfulness and transparency, explicit and legitimate purposes, data minimization (proportionality), storage period limitation, accuracy, and security—particularly confidentiality and integrity during data processing.

The French Data Protection Act (Loi Informatique et Libertés) has been amended to align with the GDPR and introduces specific provisions for processing health data, particularly in the context of research, study, or evaluation in the health field [21]. To simplify procedures, it includes reference methodologies such as MR004, which applies to studies not involving human participants [22]. It also provides reference frameworks for data controllers, offering them legal certainty in their operations. Additionally, the French National Commission for Data Protection and Liberties (CNIL) has issued guidelines for the creation and management of health data warehouses.

These guidelines define the legal and technical framework applicable to CDWs, establishing a trusted environment for the reuse of health data. They specify authorized purposes, governance principles, data categories to be processed, required security measures, and conditions for the use of CDW data in health-related research [23].

In 2022, the CNIL enhanced its recommendations by publishing a compliance checklist [24], which includes 116 criteria addressing 108 requirements. These criteria are grouped into 13 categories, with a particular focus on security and patient rights. Specifically, 56 (48.3%) criteria, representing nearly half, pertain to security measures, while 23 (19.8%) criteria focus on patient information and rights.

### Evaluation Process

Once the CDW framework was published, an exchange was initiated between institutions and the CNIL to clarify the measures required for compliance with the framework’s specifications. This process aimed to minimize the risk of misinterpretation.

In May 2023, we conducted an evaluation of the CDC’s compliance at the University Hospital of Rennes with the CNIL framework. The framework’s requirements were categorized into 2 groups: those related to the eHOP software and those pertaining to the health institution’s implementation processes. Each criterion in the compliance checklist was assessed using 1 of 3 possible values: true, false, or not applicable. A criterion was deemed not applicable if the functionality addressed by the criterion was not present in the eHOP software (eg, manual CDW feeding). A criterion was marked true if appropriate measures were implemented to meet the requirement; otherwise, it was marked false.

The value assigned to each criterion was determined by consensus between 2 assessors. For criteria where consensus could not be reached, the information security officer (ISO) and the DPO of the University Hospital of Rennes reviewed the assessment. Their input provided the definitive evaluation for those criteria. Once all criteria were assessed, final validation was conducted by both the ISO and the DPO.

This approach enabled the identification of discrepancies with the CDW framework and the determination of the overall compliance level of the CDW. Results were presented by category and expressed as the number and percentage of criteria that were not applicable, met, or not met. The overall compliance level was calculated based on all applicable criteria.

## Results

### Evaluation of Criteria Relevant to the eHOP Software

#### Overview

Of the 116 criteria in the CDW framework, 25 were identified as relevant to the eHOP software (Tables1  2), representing 22% (n=25) of the criteria. Compliance with the framework was 68% (n=15).

The results are presented in Table 2. In total, 3 of these 25 (12%) criteria were deemed not applicable to our implementation context. A total of 15 (60%) criteria were judged compliant, such as those concerning the storage of directly identifiable data in a separate space and those concerning user profiles, and 7 (28%) criteria were considered noncompliant or partially compliant and were subject to an action plan.

**Table 1.** Criteria from the clinical data warehouse framework.

Category	Criteria, n	Application eHOP, n	Institution, n
1. Target audience	1	0	1
2. Purpose	3	0	3
3. Governance	4	0	4
4. Legal basis for processing	1	0	1
5. Personal data included	12	1	11
6. Access to information	4	1	3
7. Retention period	4	4	0
8. Information for individuals—Patients	15	0	15
9.1. Information for individuals—Professionals	2	0	2
9.2. Rights of individuals	6	2	4
10. Security	56	17	39
11. Subcontractor	5	0	5
12. Transfer outside the European Union	1	0	1
13. Privacy impact assessment	2	0	2
Total	116	25	91

**Table 2.** Compliance of criteria relevant to eHOP software on May 5, 2023.

Number	Criterion	Compliance
5.2.1.1	Directly identifiable data mentioned in 5.2.1.1 are stored separately from other data	Yes
6.2	Access and use of identifiable data restricted to authorized persons for patient recontact and rights exercise	Yes
7.1 to 7.3	Data retention periods	No
7.4	Deletion or anonymization of data beyond the retention period	No
9.6	Rights of individuals: mechanism when there are no identifiable data or correspondence tables	N/A^a^
9.7	Exercise of the right to object possible permanently	Yes
10. Security		
SEC-LOG-5	Separation of profiles for access to identifiable and pseudonymized data	Yes
SEC-LOG-6.1	Separate encryption for genetic data and location data from other data	No
SEC-LOG-6.2	Encryption key for genetic data and location data accessible only by a data manager profile	No
SEC-ALI-2	Strong authentication for manual feeding of the clinical data warehouse from data entry software	N/A
SEC-PSE-1.1 to SEC-PSE-1.4	Pseudonymization of patients’ personal data	Yes
SEC-PSE-2	New pseudonymization of already pseudonymized data	Yes
SEC-PSE-3	Pseudonymization of personal data collected from health care professionals	N/A
SEC-PSE-4	Deidentification of unstructured documents	Partially
SEC-HAB-1	Different authorization profiles are provided to manage access to data as needed and exclusively	Yes
SEC-HAB-2	Granularity of access (aggregated data, pseudonymized data, directly identifiable data)	Yes
SEC-ESP-2.1	Data minimization in study datamarts	Yes
SEC-ESP-2.2	Random generation of pseudonyms for each study	Yes
SEC-ESP-3	For cohort follow-up, the same unique pseudonym can be reused across multiple workspaces	Yes
SC-JOU-1	User action logging	Yes

a N/A: not applicable.

#### Nonapplicable Criteria

The nonapplicable criteria were as follows:

- Criterion 9.6: Linkage tables enabling patient reidentification eliminate the need for additional mechanisms to respect rights. In the absence of these tables, the warehouse could be queried based on information provided by a patient to respond to their rights requests.
- Criterion 10 SEC-ALI-2: The absence of a manual feeding modality for the CDW means that no specific authentication mechanism is implemented.
- Criterion SEC-PSE-3: No PII concerning health care professionals is collected in a structured form.

#### Compliant Criteria

The eHOP CDW database is divided into multiple segregated databases, ensuring that identifying data are stored separately from medical data, in compliance with requirement 5.2.1.1 of the framework

Access rights management is based on several predefined profiles, categorized as manager and user profiles, in alignment with SEC-HAB-1 and SEC-HAB-2 requirements. The data manager profile is responsible for managing data flows from the hospital information system’s source software to the CDW, integrating data, and ensuring database quality. The study manager profile allows for the creation of datamarts for specific studies without initially including any data and manages access rights. This profile has no direct access to the CDW data. The datamart manager profile enables querying the entire CDW, except for identifying data. This user populates the datamart with query results and ensures compliance with the SEC-ESP-2.1 minimization principle by selecting only the categories of documents and information strictly necessary for the study.

User profiles are divided into 3 levels with varying access permissions. They access only data of the study datamart. Level 1 users are limited to accessing aggregated statistics; they can query the database and retrieve aggregated results but cannot view individual patient information. Level 2 users inherit level 1 permissions and can access pseudonymized documents and individual data. Level 3 users are granted access to identifying data only under exceptional circumstances, such as for pharmacovigilance professionals performing regulatory tasks. A specialized recontact manager profile has been created to meet requirements 6.2 and SEC-LOG-5. This profile allows access to a datamart’s patient list for recontact purposes. Authorized individuals with this profile cannot directly access identifying data but can activate the patient list generation function for specific studies.

The exercise of data participants’ rights is governed by institutional procedures and facilitated by a dedicated module within the eHOP CDW for managing opposition. The Medical Information Department centrally collects opposition data, which is recorded in the patient-identifying database. Rather than maintaining a “blacklist,” the system automatically excludes data from patients who have expressed opposition during the creation of datamarts, in compliance with paragraph 9.6 of the CDW framework.

Pseudonymization is performed during data integration into the CDW using a collision-free function that generates 20-byte random identifiers for patients, hospital stays, and documents. Correspondence with hospital information system identifiers is securely stored in a protected area. For each study, new pseudonyms are generated, and their correspondence with CDW identifiers is stored separately from the datamarts. All datamarts within a study share the same pseudonyms, ensuring compliance with SEC-PSE-1, SEC-PSE-2, SEC-ESP-2, and SEC-ESP-3 requirements.

Deidentification of unstructured documents is an area of ongoing improvement. Patient data deidentification has been effective since the warehouse’s inception, while health care professionals’ data are deidentified using external public databases. However, current processing times are incompatible with real-time integration of medical documents. As a result, health care professionals’ data are deidentified only in datamarts. A new algorithm, developed using machine learning methods, shows promising performance, with *F* scores ranging from 0.96 to 0.99 in initial evaluations. Its integration into the CDW would enable full compliance with the SEC-PSE-4 requirement. Access traceability is ensured through a robust logging system, with all access logs securely maintained.

#### Partially Compliant Criterion

The partially compliant criterion was as follows:

- Criterion SEC-PSE-4: The pseudonymization of health care professionals’ data in unstructured documents was not fully deployed due to incompatible processing times with real-time data integration into the CDW. However, this procedure is applied to study datamarts before making them available to users. A solution has been provided since May 2023.

#### Noncompliant Criteria

The noncompliant criteria were as follows:

- Criteria 7.1 to 7.3: Data retention periods are not yet managed in the eHOP software.
- Criteria SEC-LOG-6.1 and SEC-LOG-6.2: Implementing the functionality for encrypting and decrypting genetic data proves complex during analysis, as it requires a specific technical procedure and adapted organizational measures.

### Evaluation of Criteria Relevant to Institutions

#### Overview

Among the 116 criteria outlined in the CDW framework, 91 (78%) are under the direct responsibility of health care institutions. These criteria encompass a range of domains, including governance, patient information, individuals’ rights, and data security.

#### CDW Governance

Each institution is responsible for implementing the governance of its CDW. For instance, the University Hospital of Rennes has established governance structures such as the steering committee (comité de pilotage) and the scientific and ethical council (conseil scientifique et éthique [CSE]). The comité de pilotage determines scientific and ethical orientations and connects with other institutional bodies. The CSE provides opinions on projects requiring the use of CDW data.

#### Patient Information

The University Hospital of Rennes provides general, individual, and collective information regarding the reuse of care data for research and the implementation of the CDW through systematic notes given to patients upon their visit, posters, the welcome booklet, and the institution’s website. Additionally, the University Hospital of Rennes has collectively informed the public through press releases and awareness campaigns.

For each study based on the CDW, a note complying with Article 14 of the GDPR is posted on the institution’s website. For patients not informed about the reuse of their data and who have not revisited the institution since the CDW activation, a request for derogation from individual information was submitted to the CNIL. This derogation is required for studies involving large volumes of data where individual information is too complex and resource-intensive to implement.

#### Patient Rights

A procedure is in place to allow patients to exercise their rights. The health institution responsible for processing provides the patient with a contact point mentioned in the information note, usually the DPO. Existing procedures adopted by the institution to allow patients to exercise their rights of access and rectification of their health data are applied.

The exercise of the right to erasure (right to be forgotten) and the right to object is managed by a specific software module for managing oppositions. Once the opposition is registered, the data are flagged in the system, ensuring that they are not used in future studies. The right to erasure is treated similarly to the right to object. Patients can exercise these rights at any time.

For studies using the CDW, correspondence tables have been created to link patients’ identity data with respective studies. Thus, any unexercised right to opposition can still be applied, provided it does not compromise the study’s objectives under Article 21 of the GDPR. These guidelines regarding patient information and rights have been communicated to other institutions.

#### Security

Security requirements account for nearly half of the criteria outlined in the CDW framework. Of these, only 17 criteria are directly related to the eHOP software. For the remaining criteria, the responsibility for implementing the necessary measures to ensure compliance lies with the institution. As of May 5, 2023, 28 (72%) security criteria were deemed compliant, 3 (8%) were considered nonapplicable, and 8 (20%) were found to be noncompliant (Table 3). The overall compliance rate for security requirements stands at 77%.

**Table 3.** Criteria relevant to the institution’s information system evaluated as nonapplicable or noncompliant on May 5, 2023.

Number	Criterion	Compliance
SEC-EXP-4	Anonymous reporting of management indicators according to G29 criteria; otherwise, a reidentification risk analysis.	N/A^a^
SEC-EXP-5	Monitoring of indicator exports.	N/A
SEC-SEN-3.2	Agreement established if workstations are not under the responsibility of the controller.	N/A
SEC-ESP-1	Warehouse data are handled by researchers only in internal workspaces dedicated to each research project, isolated from the warehouse database and each other.	Partially
SEC-AUT-1.1	Access to personal data requires strong multifactor authentication.	No
SEC-AUT-2	Strong authentication for internal and external access.	No
SEC-EXP-1	Data exports outside the CDW or workspaces only if anonymized according to G29 criteria. This conformity must be documented. Otherwise, a reidentification risk analysis must be conducted and documented.	No
SEC-EXP-3.1	Exports are monitored automatically or manually by a specialized operator to verify anonymity.	No
SEC-EXP-3.2	If monitoring is automatic, noncompliant exports are flagged, quarantined, and manually verified by a trained and authorized individual.	No
SEC-JOU-2	System and network administrator access requires strong authentication and detailed access traceability, for example, using an admin bastion.	No
SEC-JOU-3	Regular control of logs, at least bimonthly, and at the end of each authorization period by an automatic monitoring solution with alert processing by an authorized operator or semiautomatic control with a manual review of abnormal logs.	No

a N/A: not applicable.

#### Nonapplicable Criteria

The nonapplicable criteria were as follows:

- Criteria SEC-EXP-4 and SEC-EXP-5: As of now, there are no dashboards for monitoring accessible from the CDW implemented at the University Hospital of Rennes.
- Criterion SEC-SEN-3.2: Workstations allowing access to the CDW at the University Hospital of Rennes are under the institution’s responsibility.

#### Partially Compliant Criterion

The partially compliant criterion was as follows:

- Criterion SEC-ESP-1: Considered partially compliant, this criterion is verified for data exploitation via the eHOP interface. Each datamart is isolated, and analyses are conducted on a secure server with controlled access. To fully comply, digital workspaces specific to each study with necessary processing and analysis tools need to be implemented for datamart exploitation.

#### Noncompliant Criteria

The noncompliant criteria were as follows:

- Criteria SEC-AUT-1.1 and SEC-AUT-2: Strong authentication was implemented in October 2023 after evaluation. This authentication concerns internal access within the institution. No external access is planned.
- Criterion SEC-EXP-1: Most studies result in publication; thus, users commit to publishing only anonymized data. They must verify the anonymity of exported results. The action plan includes revising the CDW use charter to specify this point and drafting a best practice guide for ensuring anonymity. The CSE will also verify that study protocols detail the content of the results.
- Criteria SEC-EXP-3.1 and SEC-EXP-3.2: Individual data exports are performed by CDW experts. Currently, only CDW experts conduct analyses within the CDW environment. Users commit to exporting only anonymized data.
- Criterion SEC-JOU-2: Compliance is planned.
- Criterion SEC-JOU-3: Specific alerts for the CDW have been configured since May 2023. Tools implemented by the institution, in accordance with its security policy, cover the CDW environment.

## Discussion

### Principal Findings

The framework, which is aligned with the GDPR, necessitates substantial technological and organizational adaptations to ensure compliance. Our findings highlight both the benefits and challenges associated with its implementation, offering a comprehensive perspective on its impact on CDWs.

In this discussion, we explore the study’s contributions and limitations along with the areas for improvement identified within the CDW framework. Finally, we propose measures to facilitate research projects using CDW data. These recommendations could guide regulatory authorities in future framework developments.

### Contributions and Limitations of the Study

This study is the first to evaluate the application of the CDW framework in a real-world setting within French university hospitals. The results were obtained through a rigorous evaluation process, involving dual assessments and consensus-building for all criteria. The findings were further validated by the ISO and the DPO of the University Hospital of Rennes. The proposed adjustments are grounded in practical and actionable solutions.

Our findings provide institutions with concrete insights into the challenges they face, the steps they can take to address these challenges, and potential regulatory adjustments. National authorities gain valuable field-based feedback and specific recommendations for refining the framework. Moreover, the compliance checklist proves to be an effective tool for monitoring privacy measures and could support future labeling or certification processes for CDWs.

However, the study has certain limitations. It focuses on a single institution in the French Great Western region and a single CDW technology. To generalize these findings, further studies involving diverse institutions and CDW solutions are necessary. Such studies would help uncover additional enablers and barriers to the application of the framework’s criteria.

In terms of methodology, we initially considered using a validated evaluation grid to assess the applicability of the CNIL framework. However, this approach was not adopted due to its potential for introducing bias. The CNIL framework was published after the CDW’s implementation, and a prior consultation phase with the CNIL had already defined the necessary compliance measures. Consequently, applying a retrospective evaluation grid would have been inconsistent with the timeline of the framework’s release. Instead, we opted for a descriptive approach, which effectively highlights barriers to compliance and enables the proposal of tailored, practical alternatives.

### Weaknesses of the Framework and Proposed Adjustments

Using CDW data for research requires balancing the advancement of scientific knowledge to improve patient care with the imperative to protect patient privacy. The framework imposes stringent data protection requirements, which, while essential, can hinder research efforts due to their complexity and associated costs. Our study has identified several key obstacles to the optimal use of CDW data for research purposes.

To address these challenges, regulatory adaptations could help streamline the implementation of research projects. We propose adjustments to the framework that aim to facilitate the efficient and compliant conduct of research studies and projects.

### Regarding Data Categories

#### Vital Status and Date of Death

The framework permits the collection of this information solely from medical records, which limits the CDW to recording only hospital deaths. However, the French National Institute of Statistics and Economic Studies provides a comprehensive database of deceased individuals. Several matching algorithms have been developed to use these data effectively [25-28]. Integrating French National Institute of Statistics and Economic Studies data into the CDW could address this limitation, particularly as these data are publicly available and the matching algorithms have demonstrated satisfactory performance.

#### Genetic Data

According to the CNIL, variants and mutations are classified as genetic data. The CDW framework mandates their specific encryption, which complicates their reuse despite their critical importance for personalized medicine. These data often appear in diverse document types, such as genetic reports or hospitalization records, making them challenging to isolate. We recommend limiting this encryption requirement to sequencing data exclusively, as this would simplify their management and reuse while maintaining compliance with the framework.

### Regarding Security Requirements

Some of the framework’s security requirements are already part of hospitals’ cybersecurity plans (eg, network flow segmentation, user awareness, alert setup, incident handling, and access review) [29,30]. Thus, the increasing investment of institutions in their cybersecurity should allow compliance, provided that substantial financial support is provided.

Pseudonymization is a major framework measure ensuring data confidentiality. However, this procedure does not ease regulatory steps, as pseudonymized data cannot be considered anonymous according to the European Data Protection Board [31]. Some authors argue that medical data anonymization is almost impossible [32,33], and the recommended anonymization methods are complex to implement [33]. Moreover, anonymity leads to information loss, potentially making an AI model less relevant. Greene et al [34] propose evaluating anonymity case-by-case, considering contextual factors, especially other data sources available to the user that may link back to the patient’s identity. A reidentification risk analysis, as proposed by the Health Data Hub [35], could be conducted for CDW studies by the implementation manager. If the reidentification risk is deemed minimal for a study, individual patient information could be considered nonmandatory.

The framework stipulates that only anonymized data exports are permitted. However, in research using multisource data, pseudonymized individual data exports to secure data processing platforms should be possible. The crucial point is that this environment meets security requirements. We recommend explicitly stating this possibility in the framework.

Verifying study results exported from the CDW or its workspace in aggregate form by a specialized operator seems excessive for a health care institution. Researchers’ commitment to ensuring anonymity should suffice for compliance. However, they should be supported with a best practice guide on result anonymization.

Trace control by institutions can be performed through automated control systems implemented as part of their cybersecurity plans. Integrating CDW traces into such systems is feasible, but exploiting these traces by a dedicated human operator capable of handling alerts requires additional resources that most institutions currently lack.

### Architecture Schematics

Sample architecture diagrams of the “information system” for the CDW, including workspaces, should be included in the framework. These examples would help institutions build their CDWs in line with CNIL expectations.

### Patient Information and Rights Exercise

MR004 refers to a French reference methodology for health data research that does not involve human participants. The MR004 facilitated research project implementation by waiving new individual information for each study when initial information on data reuse for research was provided to the patient, provided a transparency portal is implemented by the institution. However, initial information on CDW implementation can be challenging to deliver in practice within hospitals. One communication means to consider is the “Mon Espace Santé” platform [36], available to all citizens since February 2022. “Mon Espace Santé” is a French digital health platform where individuals can access their health data and manage their health information. Patient information could leverage this platform, similar to how organ donation opposition is integrated into the personal health record (dossier médical partagé) [37]. However, a survey by France Assos Santé, a French patient association, found that less than 50% of the population has activated their health space [38].

Public information campaigns at national, regional, and local levels would reach most of the population [39]. Collective information through a transparency portal could then replace individual study information [39].

### Exercise of Rights in CDW Studies

Current procedures allow patients to exercise their rights over integrated CDW data. However, the right to opposition and erasure for CDW studies raises questions. Once data are being analyzed, removing them seems challenging without compromising the project.

Dynamic consent and meta-consent mechanisms should be considered. Dynamic consent [40], based on transparency, allows patients to consent to each study via a dedicated platform. However, this may lead to digital divide risk and frequent patient solicitations, which could harm system acceptance, leading to default responses (consent or refusal) unrelated to the study [41].

Meta-consent [41,42] could be a more interesting solution. It involves giving prior consent to study categories based on the data nature (medical records, genomic data, etc) and research context (public or private, commercial or noncommercial, and national or international). A study by Cumyn et al [42] on meta-consent acceptability shows that the public and national nature of the processing entity, nonidentifying data, and the absence of genetic data use are factors favoring patient acceptance.

### Conclusions

This study represents the first effort to evaluate the application of the CNIL CDW framework within a real-world operational context, emphasizing its alignment with GDPR requirements. While the framework significantly enhances data security and compliance, its implementation poses practical challenges and requires substantial resources. Despite the study being limited to a single institution in France, the findings are broadly relevant, given the GDPR’s overarching influence in Europe and beyond.

The insights gained from this study are particularly pertinent, as they offer valuable lessons for other European countries and international bodies aiming to implement similar frameworks. The EHDS initiative, which seeks to create a unified framework for health data access and exchange across the European Union, can benefit from the findings of this study. The challenges and proposed adjustments highlighted here can inform the development of more efficient and scalable data governance frameworks that balance the need for rigorous data protection with the imperatives of research and innovation.

Future research should focus on expanding the scope to include multiple institutions and various CDW technologies to generalize the results and identify further facilitators or barriers to the framework’s application. Additionally, international collaborations could provide a broader perspective on best practices and innovative solutions for managing health data securely and effectively.

The importance of harmonizing data protection standards and facilitating secure data sharing cannot be overstated, especially in the context of global health crises like the COVID-19 pandemic. Effective data governance frameworks, such as the CDW framework evaluated in this study, are crucial for enabling the rapid and secure exchange of health data, which is essential for timely public health responses and ongoing medical research.

In summary, while the CDW framework provides a robust foundation for data security and compliance, its practical implementation requires careful consideration of the challenges identified in this study. By addressing these issues and fostering international cooperation, we can enhance the utility of health data warehouses, thereby supporting advancements in health care research and ultimately improving patient outcomes across the globe.

## Abbreviations

AI: artificial intelligence
CDW: clinical data warehouse
CNIL: Commission Nationale de l'Informatique et des Libertés
CSE: conseil scientifique et éthique
DPO: data protection officer
EHDS: European Health Data Space
GDPR: General Data Protection Regulation
ISO: information security officer
PII: personally identifiable information
